# Emergence, molecular mechanisms and global spread of carbapenem-resistant *Acinetobacter baumannii*

**DOI:** 10.1099/mgen.0.000306

**Published:** 2019-10-10

**Authors:** Mohammad Hamidian, Steven J. Nigro

**Affiliations:** ^1^​ The ithree institute, University of Technology Sydney, Ultimo, NSW 2007, Australia; ^2^​ Communicable Diseases Branch, Health Protection NSW, St Leonards, NSW 2065, Australia

**Keywords:** *Acinetobacter baumannii*, global clones, GC1, GC2, carbapenem resistance, *oxa23*, *oxa58*, *oxa24*, *oxa235*, Tn*2006*, Tn*2008*, Tn*2009*, AbaR4 and plasmid

## Abstract

*
Acinetobacter baumannii* is a nosocomial pathogen that has emerged as a global threat because of high levels of resistance to many antibiotics, particularly those considered to be last-resort antibiotics, such as carbapenems. Although alterations in the efflux pump and outer membrane proteins can cause carbapenem resistance, the main mechanism is the acquisition of carbapenem-hydrolyzing oxacillinase-encoding genes. Of these, *oxa23* is by far the most widespread in most countries, while *oxa24* and *oxa58* appear to be dominant in specific regions. Historically, much of the global spread of carbapenem resistance has been due to the dissemination of two major clones, known as global clones 1 and 2, although new lineages are now common in some parts of the world. The analysis of all publicly available genome sequences performed here indicates that ST2, ST1, ST79 and ST25 account for over 71 % of all genomes sequenced to date, with ST2 by far the most dominant type and *oxa23* the most widespread carbapenem resistance determinant globally, regardless of clonal type. Whilst this highlights the global spread of ST1 and ST2, and the dominance of *oxa23* in both clones, it could also be a result of preferential selection of carbapenem-resistant strains, which mainly belong to the two major clones. Furthermore, ~70 % of the sequenced strains have been isolated from five countries, namely the USA, PR China, Australia, Thailand and Pakistan, with only a limited number from other countries. These genomes are a vital resource, but it is currently difficult to draw an accurate global picture of this important superbug, highlighting the need for more comprehensive genome sequence data and genomic analysis.

## Data Summary

1. Three thousand five hundred and seventy-five *A. baumannii* genomes were retrieved from the GenBank non-redundant and Whole Genome Shotgun (WGS) databases and analysed here. The full strain list and the ftp addresses used to retrieve the genomes are publicly available at https://www.ncbi.nlm.nih.gov/genome/?term=Acinetobacter+baumannii.

2. Variants of the beta-lactam resistance genes used for analyses were retrieved from the NCBI Antimicrobial Resistance Reference Gene database, which is publicly available at https://www.ncbi.nlm.nih.gov/pathogens/isolates#/refgene/.

**Impact Statement**

Carbapenem antibiotics were once considered to be a last resort, but the rapid worldwide dissemination of multiply antibiotic-resistant (MAR) bacteria has made them the first, or only, treatment option left for many infections. However, organisms that are also resistant to carbapenems are now becoming commonplace. Diverse populations of carbapenem-resistant *Acinetobacter baumannii* (CRAB) have been observed worldwide, mainly driven by the spread of two MAR clonal lineages. Next-generation sequencing technologies have provided an unprecedented level of information to study the evolution and epidemiology of carbapenem resistance in this priority pathogen. Here, we delve into this rich resource to not only enhance what is known about the mechanisms and epidemiology of carbapenem resistance in *A. baumannii *, but also understand what is missing. Understanding the factors that lead CRAB to spread so successfully throughout the world is crucial to curtail its spread and prevent it from becoming universally untreatable.

## Introduction

Antibiotic resistance has increased to dangerously high levels in bacterial strains recovered in all parts of the world, threatening our ability to treat common infectious diseases [1]. *Acinetobacter baumannii* is one such organism and a member of the ESKAPE group of six bacterial pathogens (* E* *nterococcus faecium *, *Staphylococcus aureus *, *Klebsiella pneumoniae *, *Acinetobacter baumannii *, *Pseudomonas aeruginosa* and *Enterobacter* species) that are major causes of antibiotic-resistant infections [2]. *A. baumannii* is a Gram-negative opportunistic nosocomial pathogen that is most notably responsible for pneumonia, along with infections of burns and other wounds [3–5]. It can survive harsh environmental pressures, such as desiccation and pH extremes, making management of these infections particularly challenging in the intensive care and burns units of hospitals [6].

*A. baumannii* has been recognized as a threat since the 1970s [4, 7] due to the rapid development of resistance to a wide range of antibiotics, including last-resort treatments such as carbapenems [8–11]. Often, there are very limited or no remaining options to treat *A. baumannii* infections [3, 12]. In 2017, this prompted the World Health Organization (WHO) to recognize carbapenem-resistant *A. baumannii* (CRAB) as the critical, number 1 priority among a published list of 12 antibiotic-resistant bacteria that pose the greatest threat to modern medicine, underlining the clinical significance and global burden of infections caused by CRAB [13].

Here, we discuss the emergence, molecular mechanisms and global spread of CRAB. To develop a snapshot of the geographical distribution of genomes sequenced so far and their carbapenem resistance gene (CRG) repertoire, we explore over 3500 *A. baumannii* genomes deposited in the GenBank non-redundant and Whole Genome Shotgun (WGS) databases. We also examine the genomic context of CRGs in all 128 complete genomes to further understand the role of mobile genetic elements in the spread of CRGs in *A. baumannii *.

## Global spread of carbapenem-resistant *Acinetobacter baumannii*

Carbapenem antibiotics such as meropenem and imipenem belong to the ß-lactam family and remain active against most ß-lactamase-producing organisms, including those with extended spectrum ß-lactamase enzymes [14]. Carbapenems are considered to be a front-line treatment for infections caused by multiply resistant bacteria [15], but carbapenem resistance is increasingly common in *A. baumannii *, imposing huge financial and healthcare burdens [8, 16–18].

The number of studies in PubMed reporting CRAB increased from a single report [19] in 2000 to over 266 in 2018, highlighting its global dissemination [20, 21]. This has been largely due to inter- and intra-hospital transfer of resistant strains over the last two decades [21–23]. One classic example involving both intra-hospital and international transfer was the dramatic increase in *A. baumannii* infections in soldiers injured in war zones in Iraq and Afghanistan between 2006–2008 [24–28]. These infections often were resistant to multiple antibiotics and one study showed that 37 % of isolates recovered from injured deployed military personnel were also resistant to carbapenems [29]. A subsequent study found that isolates recovered from injured soldiers were genetically related to those recovered on field hospital surfaces rather than pre-injury colonization or introduction at the time of injury [28]. It has been suggested that the return of soldiers from combat zones was an important factor that contributed to the epidemiology of *A. baumannii* infections in the USA [21].

Outbreaks caused by CRAB have been reported from civilian hospitals in the USA, Canada, South America, Europe, Africa, the Middle East, Southeast Asia, Australia and many more countries [11, 18, 23, 30–72]. Generally, these CRAB outbreaks have been caused by the spread of a few specific clones that were already resistant to a wide range of antibiotics [22, 41, 73]. Although it was initially thought that these clones were limited to Europe [43, 74], they have now been reported in different countries of all inhabited continents [16, 18, 75–86], raising widespread clinical concerns [9, 16, 56, 59, 78, 79, 83–90]. The two major clones responsible for most of these outbreaks are now commonly referred to as global clone 1 (GC1) and global clone 2 (GC2), but have also been referred to as international clones 1 and 2 [8, 16, 75, 78, 90, 91].

**Fig. 1.**
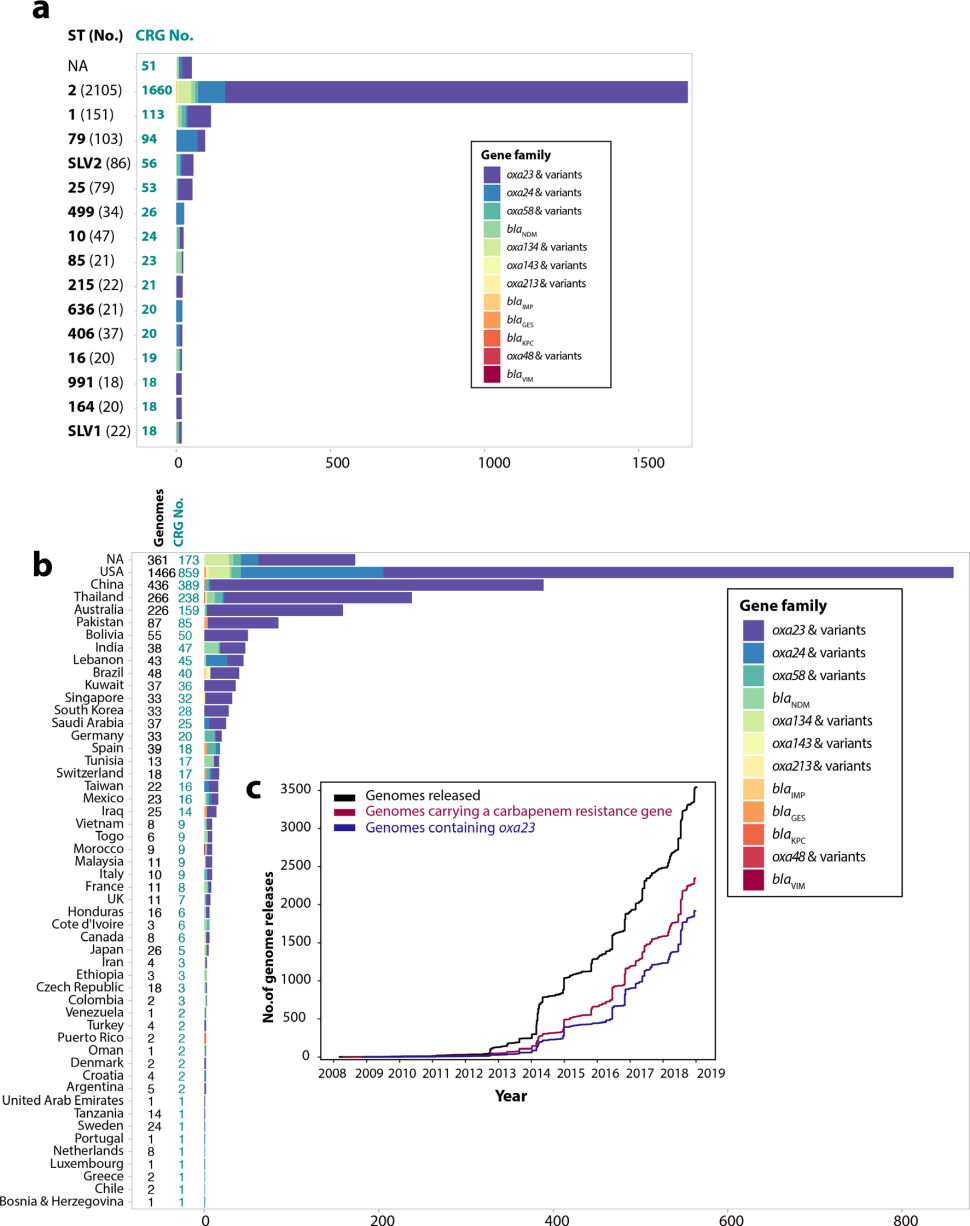
Distribution of carbapenem resistance genes and trend of *A. baumannii* genomes released. (a) Distribution of carbapenem resistance genes in the 15 most prevalent sequence types (STs; according to the Institut Pasteur MLST scheme). Numbers coloured turquoise indicate carbapenem resistance genes and black numbers show STs. SLV1 and SLV2 indicate single-locus variants of ST1 and ST2, respectively. All STs are based on the Institut Pasteur MLST scheme. (b) Geographical distribution of CRGs in *A. baumannii* genomes publicly available in the GenBank non-redundant and WGS databases (only countries with ≥1 CRG-containing genome are shown). Countries are shown on the *y*-axis and the numbers on *x*-axis indicate the number of CRGs. (c) *Acinetobacter* genomes released between 2008 and early April 2019. Black indicates total genome releases, red shows genomes with a carbapenem resistance gene and dark purple indicates genomes carrying the *oxa23* gene. These figures were drawn using the ggplot2 package in R v3.5.2.

As of early April 2019, there were 3609 *A. baumannii* genomes available in the GenBank non-redundant and WGS databases (https://www.ncbi.nlm.nih.gov/genome/?term=Acinetobacter+baumannii). Here, these genomes were downloaded, and MLST types were determined *in silico* using MLST v2.16.1 (https://github.com/tseemann/mlst) followed by screening for antibiotic resistance genes using Abricate v0.8.10 (https://github.com/tseemann/abricate). These data were combined with the metadata available for each genome using R v3.5.2. Thirty-four duplicate, or passaged, isolates were removed from the analysis. Of the 3575 remaining genomes analysed here, 2364 (66 %) were members of GC1 (173 genomes) and GC2 (2191 genomes). These clones are defined here as ST1, representing GC1s according to the Institut Pasteur MLST scheme [92], and ST2, representing GC2s, along with their single-locus variants (ST1, SLV1, ST2 and SLV2 in Fig. 1a). However, ST2 itself is by far the dominant type, with 2105 genomes (59 %) among the available complete and draft genomes (Fig. 1a). This is also consistent with a large number of previous publications that continue to report outbreaks due to these two global clones, with GC2s accounting for the bulk of CRAB outbreaks [41, 43, 56, 77, 80, 81, 84, 85, 87, 93–96]. The global distribution of CRAB has been heavily influenced by the spread of GC2 isolates, with 1678 GC2 isolates also carrying at least 1 CRG. Only 109 of the 3575 genomes were GC1 isolates that harboured at least 1 CRG. Two other lineages contributed almost as much as GC1, with 91 and 53 belonging to ST79 and ST25, respectively.

## Molecular mechanisms of carbapenem resistance in *A. baumannii*

Many carbapenem resistance mechanisms have been described in *A. baumannii *, including alterations or loss of outer membrane proteins such as CarO [97, 98] and modifications of the AdeABC resistance nodulation division (RND) efflux pump [99, 100]. Although efflux modifications contribute to carbapenem resistance in *A. baumannii *, on their own they are are not sufficient to cause clinically relevant resistance [100]. Carbapenem resistance in *A. baumannii* is largely due to the horizontal acquisition of genes that encode carbapenem-hydrolyzing enzymes belonging to either Ambler class D (oxacillinases) or class B (metallo-ß-lactamases) [101–103].

### Oxacillinases

Oxacillinase enzymes (OXAs) are a heterogeneous family [104] and, to date, several groups of carbapenem-hydrolyzing oxacillinases have been described in *A. baumannii *, most notably OXA-23, OXA-24, OXA-58, OXA-143, OXA-235 and an intrinsic OXA [105, 106], designated OXA-Ab for simplicity [89]. Genes encoding these acquired carbapenem-hydrolyzing enzymes are the main cause of carbapenem resistance in *A. baumannii* [107]. Generally, oxacillinases only hydrolyze carbapenems weakly and are often poorly expressed, hence they cannot cause clinically relevant levels of resistance on their own [108]. However, their expression is often enhanced by the insertion of an upstream IS, which enhances expression by providing a strong promoter, causing high resistance levels [109–111]. Reports of the prevalence of these genes vary by geographical distribution, but *oxa23* is the most frequently described [34, 35, 47, 49, 50, 55, 58, 63, 66, 67, 75, 77, 106, 112, 113].

#### The intrinsic *oxaAb*

The *oxaAb* gene (also known as *bla*_OXA51-like_) occurs naturally in *A. baumannii* and is used as a marker for speciation [114]. So far, over 180 *oxaAb* variants (https://www.lahey.org/studies/) have been identified in *A. baumannii* strains [115–117]. The most common variants are *oxa6*9 and *oxa6*6, which are associated with members of GC1 and GC2, respectively [78, 89, 90]. It has been suggested that strains with an ISAba1 upstream of *oxaAb* can be carbapenem-resistant [109], but more than just overexpression of *oxaAb* is needed for significant levels of carbapenem resistance [118]. However, further work is required to understand the contribution of *oxaAb* overexpression in different genetic backgrounds and whether specific amino acid alterations in OXA-Ab also play a significant role.

#### Acquired oxacillinases

The *oxa23* gene was first characterized in an *A. baumannii* strain recovered in Scotland in 1985, shortly after the introduction of carbapenems as therapeutic agents [119, 120]. Later, it was shown to have originated from the chromosome of *Acinetobacter radioresistens *, where it was mobilized into *A. baumannii* by ISAba1 [121]. To date, more than 25 variants of *oxa23* have been identified (https://www.ncbi.nlm.nih.gov/pathogens/isolates#/refgene/BETA-LACTAM).

The *oxa58* gene was first identified on a plasmid from a multiply antibiotic-resistant *A. baumannii* recovered in France in 2003 [122], and to date six further variants have been found. The *oxa58* gene has been associated with hospital outbreaks in Europe, the USA, South America, Australia and Africa [37, 39, 104, 105, 107, 123].

The *oxa24* gene was originally identified in the chromosome of a CRAB isolate recovered in Spain [105]. However, now CRAB strains carrying the *oxa24* gene*,* and its variants, often on small plasmids, have been recovered in hospital outbreaks worldwide [19, 53, 65, 105, 124–128]. This oxacillinase group consists of eight close relatives and amongst them OXA-25, OXA-26, OXA-40/OXA-24 (a sequencing error initially misclassified these as different) and OXA-72 are the most prevalent variants [129].

Other class D carbapenemases, such as those belonging to the OXA-143 and OXA-235 families, have also been associated with CRAB outbreaks in several countries [130], although they are reported less frequently and are generally considered to be minor causes for carbapenem resistance in *A. baumannii *.

### Other carbapenemases

Other CRGs, such as those encoding metallo-ß-lactamases (MBL), *bla*_VIM_, *bla*_IMP_ and *bla*_NDM_, or class A carbapenemases, *bla*_KPC_ and *bla*_GES-11_, are also seen in *A. baumannii* [45]. However, unlike Enterobacteriaceae [131], they are not common in *A. baumannii* [45]. The *bla*_NDM_ gene is often located in a mobile 10 kb ISAba125-bounded composite transposon called Tn*125* [132], which is commonly seen on conjugative plasmids [133, 134].

## Genome sequencing; opportunities and challenges

In recent years, whole-genome sequencing (WGS) technologies and advances in bioinformatic tools have revolutionized the study of bacterial pathogens, enabling gene screening and phylogenomic studies of outbreak strains with unprecedented resolution [135–137]. The first *A. baumannii* genome was sequenced in 2006, ATCC 17978 [138], followed by an epidemic GC1 strain in France, a non-clonal strain from human body lice [139] and a carbapenem-resistant GC2 strain recovered in Italy [140]. A few additional strains, including three CRAB GC1 strains (AB0057, AB056 and AB059) recovered from military patients at Walter Reed Army Medical Center, were also sequenced in the USA between 2008–2010 [9, 16]. However, as short-read sequencing technologies became more affordable and accessible, the number of genomes sequenced exponentially increased from 2014 onwards, and by early April 2019, over 3500 *A. baumannii* genomes were available (Fig. 1c).

**Fig. 2.**
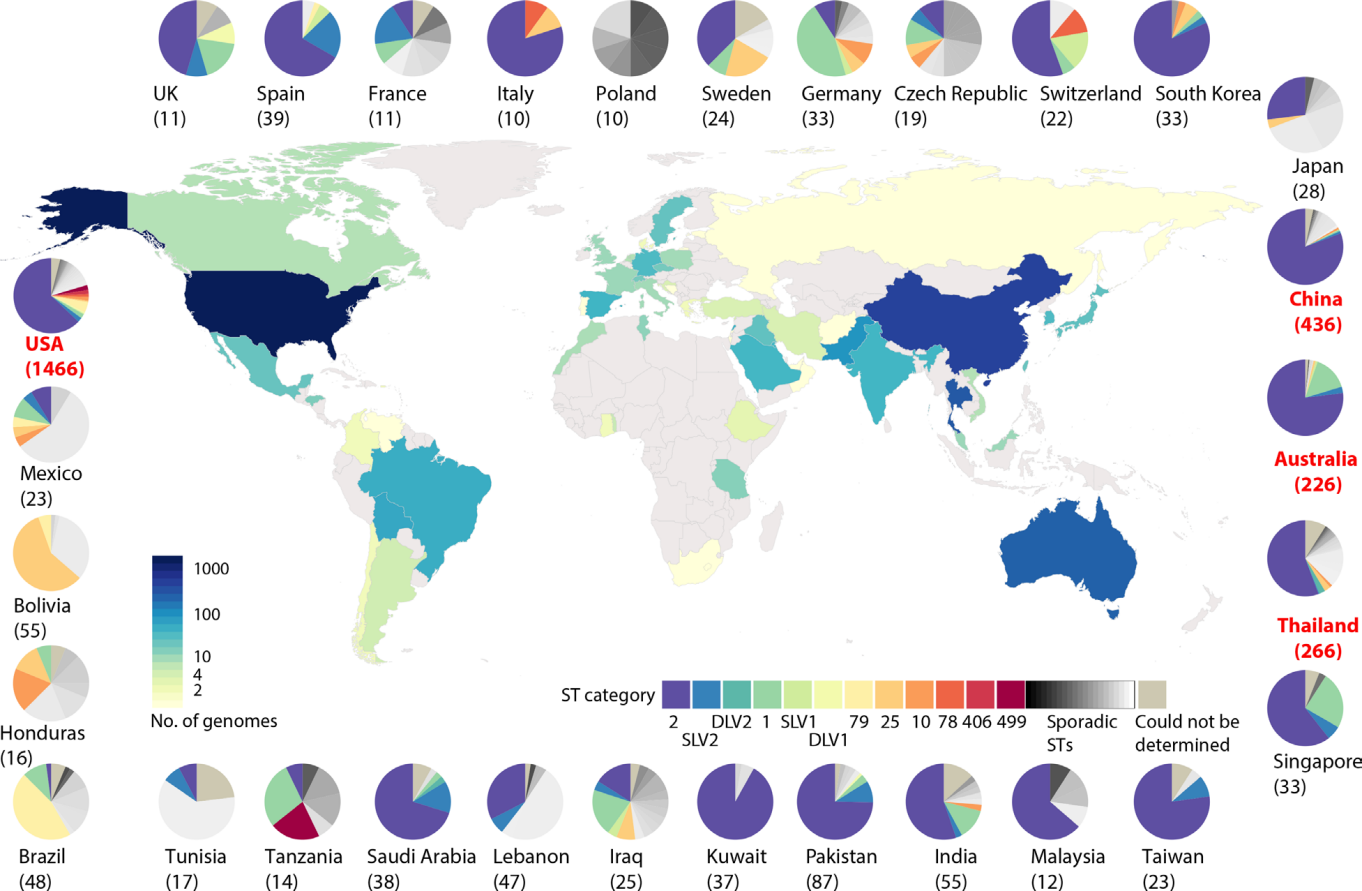
Geographical distribution of *A. baumannii* genomes released. Countries are colour coded according to the number of genomes available as of April 2019. Countries with no genome available are coloured grey. Pie charts indicate the distribution of STs in each country. Sequence types (STs) were determined according to the Institut Pasteur MLST scheme.

Amongst the 3575 non-redundant genome sequences studied here, 2345 (66 %) contained at least one CRG and of these, 1918 genomes (82 %) carry at least 1 copy of *oxa23* (Fig. 1b), consistent with worldwide reports that *oxa23* is overwhelmingly predominant in *A. baumannii* [34, 47, 49, 50, 55, 58, 63, 66, 77]. However, these sequences have a skewed geographical distribution, with 69 % (2481/3575) of all sequenced strains isolated from only five countries, namely the USA, PR China, Thailand, Australia and now Pakistan (Fig. 2). Notably, a total of 57 % (838/1466), 89 % (387/436), 84 % (224/266), 70 % (159/226) and 93 % (81/87) of genomes sequenced from the USA, PR China, Thailand, Australia and Pakistan, respectively, carry a CRG of one kind (Fig. 1b). However, it is unclear to what extent these CRG proportions reflect true population trends in these countries or whether they are a result of preferential selection of CRAB isolates for large-scale WGS projects, highlighting the need to sequence all *A. baumannii* that cause infection, regardless of their resistance phenotype. Hence, it is difficult to draw a clear picture of the true global *A. baumannii* population, including CRAB, unless more representative strains from Europe, the Middle East, Russia and Africa are sequenced and made publicly available.

Furthermore, whilst GC1 and GC2 are the most common clones in many countries, this may not always be true. For instance, GC1 and GC2 strains do not seem to be the dominant types in South American countries (*n*=10/70), Tunisia (*n*=1/13), Tanzania (*n*=5/14), Poland (*n*=0/10) and Japan (*n*=7/26) (Fig. 2), although more genomes are needed to confirm this. Although GC2 appears to be the dominant type in some countries, for example Spain, the genomes show that *oxa24* and *oxa58* are the dominant CRGs rather than *oxa23* (Fig. 2). However, given the relative paucity of genome data from these countries, caution needs to be exercised when drawing such conclusions.

Studying the genetic environment of antibiotic resistance genes, including CRGs, often provides valuable information on the origin, emergence, evolution and spread of resistance throughout bacterial populations [141]. Most of the currently available *A. baumannii* genomes have been sequenced using short-read technologies such as Illumina HiSeq or MiSeq. Although the data produced by these methods are sufficient to identify antibiotic resistance genes or draw phylogenetic trees, they lack the power to resolve complex resistance regions, which are often made up of numerous repeated elements [8, 142]. These regions tend to compromise assembly and can only be resolved manually via PCR and Sanger sequencing or by using long-read sequencing technologies such as Oxford Nanopore Technology (ONT) or Pacific Biosciences (PacBio) [143, 144]. Indeed, only 128 (4 %) genomes have been fully assembled and the majority of these were sequenced with PacBio (data not shown).

## Genomic contexts and the role of mobile genetic elements in the spread of carbapenem resistance genes

**Fig. 3.**
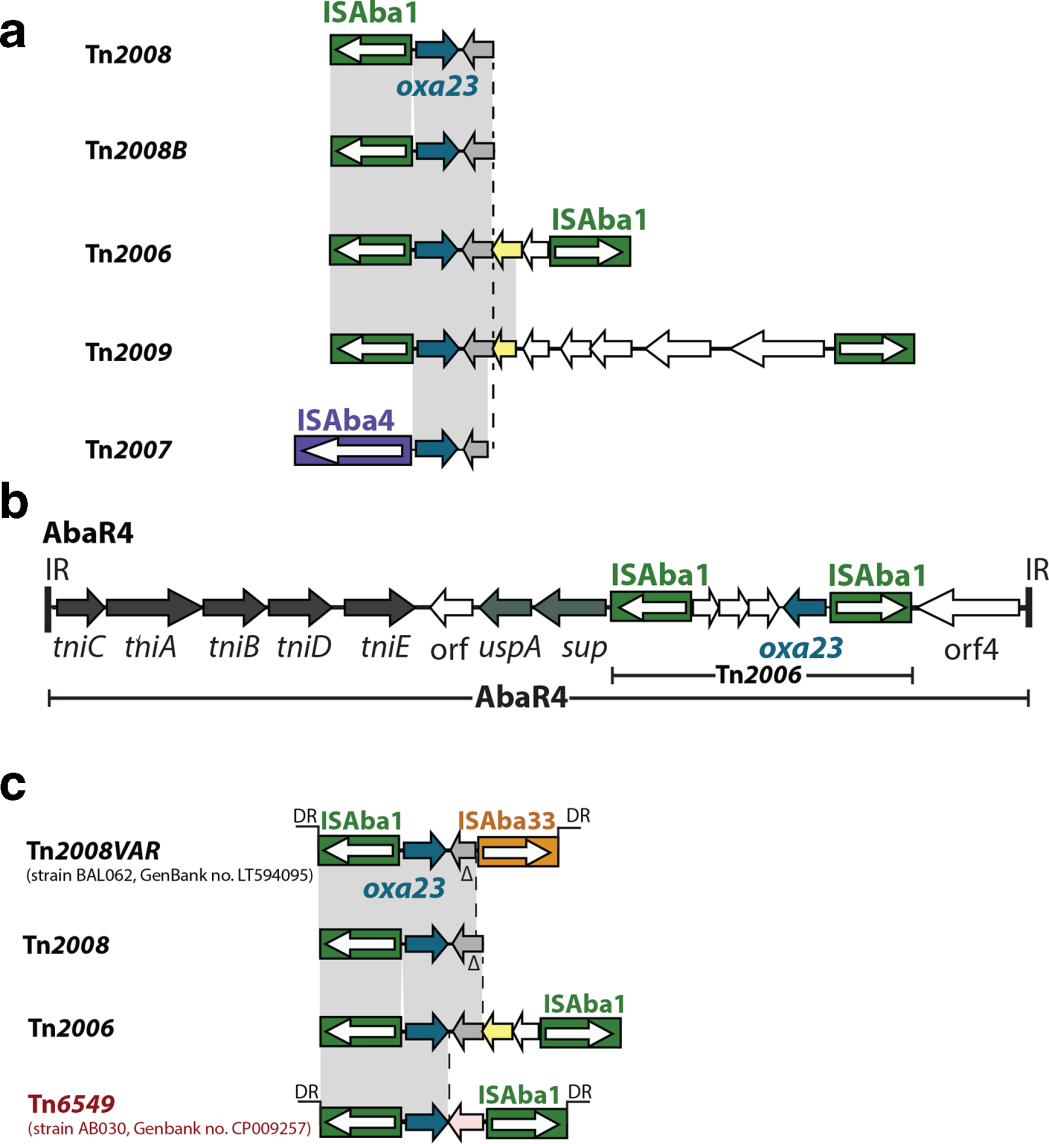
Structure of transposons carrying the *oxa23* gene. (a) Genes and open reading frames are shown using arrows. Filled boxes are insertion sequences (ISs) with ISAba1 coloured green, ISAba33 coloured dark orange and ISAba2 coloured dark purple. Arrows inside the boxes indicate the direction of transposition gene expression. The *oxa23* gene is shown in dark blue and open reading frames encoding hypothetical proteins are shown in white. (b) Vertical bars marked as IR indicate inverted repeats of AbaR4. (c) DRs indicates direct repeats. Arrows located in the central segments of Tn*2006*, Tn*2008* and Tn*6549*, coloured grey, yellow and pink, respectively, indicate open reading frames that encode unrelated hypothetical proteins.

The *oxa23* gene has moved into chromosomes and plasmids, on multiple occasions, via the transposons Tn*2006*, Tn*2007*, Tn*2008*, Tn*2008B*, Tn*2009* and AbaR4 (Fig. 3a) [8, 9, 18, 112, 113, 145–148]. The *oxa23*-containing Tn*2006* is the most commonly found transposon, and hence the most important in CRAB [110, 112]. It is a 4.8 kb class I transposon that consists of a central 2445 bp segment bounded by two inversely oriented copies of ISAba1 and generates a 9 bp target site duplication (TSD) upon insertion [110], characteristic of ISAba1 transposition [111]. Tn*2006* can move independently and is found in many different chromosomal and plasmid contexts in distantly related *A. baumannii* strains [113]. In members of a distinct clade within GC1 lineage 1, Tn*2006* has been found in a specific chromosomal location [8, 9, 18]. Tn*2006* within AbaR4 is also located in the chromosomal *comM* gene in a member of another GC1 lineage, lineage 2 [70], where the *A. baumannii* Resistance Island (AbaR) is often present [149]. In GC2 isolates, *oxa23* is often found in Tn*2006* alone or in derivatives of AbaR4 as components of *A. baumannii* Genomic Resistance Island (AbGRI), which resides in the same location in *comM* as AbaR does in GC1s [11, 112]. Tn*2008,* Tn*2008B* and Tn*2009* (Fig. 3 and Table S1, available in the online version of this article) are also seen in several chromosomal positions and are not associated with genomic islands [112].

Plasmids also play a crucial role in the spread of multiple carbapenem resistance genes in *A. baumannii* [45, 112, 130, 150]. For instance, large conjugative plasmids (80–130 kbp) encoding the RepAci6 replication initiation protein [71] are implicated in the spread of *oxa23* in GC1 and GC2, as well as strains that do not belong to these clones [71, 91, 151]. To date, several *oxa23* transposons have been found in different locations of related RepAci6 plasmids [71, 91, 146] . Moreover, *oxa23* in Tn*2006* was recently found in a RepAci1 plasmid where it was shown to be mobilized by a RepAci6 plasmid [152], further emphasizing the role of plasmids encoding RepAci6 in spreading and now in the mobilization of carbapenem resistance between disparate strains.

Currently, there are 128 completed *A. baumannii* genomes in GenBank, of which 91 carry at least one CRG. Examining these 91 genomes shows that 9 of the 11 *oxa23*-containing GC1s (Table S1) carry *oxa23*, either in Tn*2006* in the chromosome or, in one case, Tn*2006* in AbaR4 in *comM* [70]. In several strains, AbaR4 is found in a RepAci6 plasmid (Table S1). Members of GC2 often carry Tn*2006* in the chromosome as part of AbGRI variants, with several strains carrying two *oxa23* copies in this island (Table S1). Four strains, two from the Republic of Korea and two from Pakistan (Table S1), carried Tn*2008B* (Fig. 3a) in different chromosomal positions flanked by different TSDs. This suggests that Tn*2008B* is still quite active, as it was chromosomally incorporated on multiple occasions. Other GC2s carry either Tn*2006* or Tn*2008* in variants of RepAci6 plasmids (Table S1). Tn*2009* appears most commonly in GC2 isolates from PR China (*n*=17) and the Republic of Korea (*n*=19), some with multiple chromosomal copies in tandem (Table S1). In non-GC1 or GC2 strains, chromosomal Tn*2006* appears most often. Interestingly, 39 complete genomes harbour more than 1 copy of *oxa23*. Most often, there are multiple copies in the chromosome, although some also have *oxa23* on a plasmid. This raises the question of whether these isolates, or those that harbour multiple families of CRG, have a selective advantage compared to those with fewer copies of *oxa23*, and warrants further investigation.

New *oxa23*-containing structures are still being identified, such as an ISAba1- and ISAba33-flanked transposon (Fig. 3c) described in 2016 [153]. Indeed, during the course of this work we found *oxa23* in yet another novel structure with the features of a composite transposon, in the chromosome of a Canadian strain (BA30 in Fig. 3c). This 3902 bp transposon contains *oxa23* flanked by directly oriented copies of ISAba1. Two copies of this transposon were found at different chromosomal locations (bases 3303155–3307056 and 4295797–4299698 in CP009257), with each copy flanked by novel 9 bp TSDs, providing evidence that it moves independently. Hence, we named this transposon Tn*6549* (Fig. 3c). Tn*6549* appears to be a derivative of Tn*2008,* rather than Tn*2008B*, as there is 27 bp between the start of the *oxa23* gene and the ISAba1 sequence, which is indicative of Tn*2008* [112]. The central segment of Tn*6549* contains *oxa23* and an open reading frame of unknown function (orf in Fig. 3c). A sequence identical to this open reading frame was found in the chromosome of *Acinetobacter* sp. strain ACNIH1 (GenBank accession no. CP026420) and fragments of this open reading frame are in several *Acinetobacter lwoffii* and *Acinetobacter haemolyticus* plasmids (e.g. GenBank accession nos CP038010 and CP032112).

The *oxa58* gene is often embedded in the ISAba3::ISAba2-*oxa58*-ISAba3 structure, and carried on non-conjugative plasmids encoding both RepAci1 and RepAci10 [107, 140, 154]. This entire structure is now known to be surrounded by short inversely oriented inverted repeats similar to chromosomal *dif* sites, now referred to as p*dif*, targeted by XerC–XerD site-specific recombinases [154–156]. This *dif* module containing *oxa58* is found in different plasmid backgrounds, indicating that it is a discrete mobile element that is responsible for the movement of *oxa58,* rather than the ISs that surround it [154–156]. Analysis of the completed genomes also indicated that *oxa58* is mainly associated with *dif* modules often carried by similar small plasmids encoding RepAci1 and RepAci10 (Table S1).

The *oxa24* gene is commonly seen in 8–12 kb plasmids that encode RepAci1 or RepAci2, as part of discrete *dif* modules flanked by p*dif* sites [155, 157–159]. This was also the case in all complete genomes with *oxa24,* (Table S1), adding further evidence that small plasmids, particularly those encoding RepAci1 and RepAci2, are a major force behind the global spread of *oxa58* and *oxa24*.

The *oxa253* gene, a variant of *oxa143,* may also occur in p*dif* modules, as it has been found near a single p*dif* site in a context similar to *oxa24* in a RepAci2 plasmid [160]. The *oxa235* gene, and its variants *oxa236* and *oxa237*, are often found in single-nucleotide variants of a 5.2 kb ISAba1-bounded composite transposon called Tn*6252*, which has been found in chromosomes and plasmids [161–163].

### Conclusions

Antibiotic resistance is on the rise and we are already running out of antibiotics to treat CRAB, which are unfortunately most commonly resistant to a wide range of additional antibiotics. Members of GC1 and GC2 are responsible for the bulk of globally disseminated multi-resistant *A. baumannii *, including CRAB. Although the current publicly available genomes provide an invaluable snapshot of the evolution and spread of CRAB throughout much of the world, the paucity of publicly available genome sequence data from regions such as Europe, the Middle East, Russia, Africa and South America has made it difficult to draw an accurate global picture of the spread of *A. baumannii* clones, CRGs and their phylogeny. Notably, whilst *oxa23* is predominant globally, this is not always the case, particularly in countries such as Spain, Germany or Tunisia (Fig. 1b), further emphasizing the need for more sequencing coverage to understand why these different CRGs dominate in different regions. Sequencing strains from diverse regions is vital in understanding the evolutionary trajectory of the two major global clones, as well as other emerging clones, such as ST79 or ST25.

Expanding the use of long-read sequencing will facilitate a better understanding of the mobile elements responsible for moving CRGs and their broader contexts, while also enabling the characterization of further novel transposons and conjugative and mobilizable plasmids. It is now clear that alternative methods of horizontal gene transfer, such as *dif* modules and homologous recombination, play a larger role in the dissemination of CRGs than previously thought. Completing genome and plasmid assemblies will provide further knowledge regarding how widespread and important these mechanisms truly are. Indeed, this understanding will be vital in curtailing the future spread of CRGs in *A. baumannii *, as the issues of CRG spread via successful strains or clones and the spread of CRGs to susceptible strains via HGT are distinct problems that require different solutions. This will be crucial to identify molecular and epidemiological diagnostic markers to help identify resistant clones and track their spread. A more geographically uniform distribution of genome sequence data is also needed to further monitor plasmid movement and identify the true proportion of *A. baumannii* harbouring conjugative plasmids carrying *bla*_NDM_ and other CRGs that are common in other species. With the lack of new antibiotics to treat CRAB, and the uncertainty about whether new drugs would even be effective, infection control policy and practice built upon the framework of these phylogenetic and epidemiological analyses are vital in stopping the spread of CRAB. Without such interventions, we will enter an era where common infections and minor injuries caused by CRAB can once again kill.

## Data bibliography

1. GenBank non-redundant and Whole Genome Shotgun (WGS) databases. Complete ftp addresses used to retrieved the genomes are publicly available at https://www.ncbi.nlm.nih.gov/genome/?term=Acinetobacter+baumannii.

2. National Centre for Biotechnology Information (NCBI) Antimicrobial Resistance Reference Gene database, publicly available at https://www.ncbi.nlm.nih.gov/pathogens/isolates#/refgene/.

## Supplementary Data

Supplementary File 1
